# NCO-sP(EO-*stat*-PO) Coatings on Gold Sensors—a QCM Study of Hemocompatibility

**DOI:** 10.3390/s110505253

**Published:** 2011-05-13

**Authors:** Stefan Sinn, Mirjam Eichler, Lothar Müller, Daniel Bünger, Jürgen Groll, Gerhard Ziemer, Frank Rupp, Hinnak Northoff, Jürgen Geis-Gerstorfer, Frank K. Gehring, Hans P. Wendel

**Affiliations:** 1 Clinical Research Laboratory, Department of Congenital & Pediatric Cardiac Surgery, Children’s University Hospital, Tuebingen University, Calwerstr 7/1, 72076 Tuebingen, Germany; E-Mails: stefan.sinn@thg-lab.de (S.S.); gd.ziemer@uni-tuebingen.de (G.Z.); 2 Department of Prosthetic Dentistry, Section Medical Materials and Technology, Tuebingen University, Osianderstrasse 2-8, 72076 Tuebingen, Germany; E-Mails: mirjam.eichler@med.uni-tuebingen.de (M.E.); frank.rupp@uni-tuebingen.de (F.R.); juergen.geis-gerstorfer@uni-tuebingen.de (J.G.G.); 3 Biosensor Research Group, Institute of Clinical and Experimental Transfusion Medicine, Tuebingen University, Germany; E-Mails: lothar.mueller.med@googlemail.com (L.M.); hinnak.northoff@med.uni-tuebingen.de (H.N.); frank.gehring@med.uni-tuebingen.de (F.K.G.); 4 DWI e.V. and Institute of Technical and Macromolecular Chemistry, RWTH Aachen University, Pauwelsstr. 8, D-52056 Aachen, Germany; E-Mails: buenger@dwi.rwth-aachen.de (D.B.); groll@fmz.uni-wuerzburg.de (J.G.); 5 Department of Functional Materials in Medicine and Dentistry, University Hospital Wuerzburg, Pleicherwall 2, D-97070 Wuerzburg, Germany

**Keywords:** surface coating, biosensors, hemocompatibility, QCM, protein adsorption

## Abstract

The reliability of implantable blood sensors is often hampered by unspecific adsorption of plasma proteins and blood cells. This not only leads to a loss of sensor signal over time, but can also result in undesired host *vs*. graft reactions. Within this study we evaluated the hemocompatibility of isocyanate conjugated star shaped polytheylene oxide—polypropylene oxide co-polymers NCO-sP(EO-*stat*-PO) when applied to gold surfaces as an auspicious coating material for gold sputtered blood contacting sensors. Quartz crystal microbalance (QCM) sensors were coated with ultrathin NCO-sP(EO-*stat*-PO) films and compared with uncoated gold sensors. Protein resistance was assessed by QCM measurements with fibrinogen solution and platelet poor plasma (PPP), followed by quantification of fibrinogen adsorption. Hemocompatibility was tested by incubation with human platelet rich plasma (PRP). Thrombin antithrombin-III complex (TAT), β-thromboglobulin (β-TG) and platelet factor 4 (PF4) were used as coagulation activation markers. Furthermore, scanning electron microscopy (SEM) was used to visualize platelet adhesion to the sensor surfaces. Compared to uncoated gold sensors, NCO-sP(EO-*stat*-PO) coated sensors revealed significant better resistance against protein adsorption, lower TAT generation and a lower amount of adherent platelets. Moreover, coating with ultrathin NCO-sP(EO-*stat*-PO) films creates a cell resistant hemocompatible surface on gold that increases the chance of prolonged sensor functionality and can easily be modified with specific receptor molecules.

## 1. Introduction

Biosensors consist of biological sensitive elements like enzymes, peptides, antibodies or aptamers [1], and transducer elements that transform the signal from the specific interaction between the recognition element and the analyte into a signal that can be measured by associated electronics. Besides the technical aspects of the selection and modification of different transducers and electronics, coating and immobilization techniques of biological recognition elements to the sensor surface are a field of continuous research and development.

According to their applications biosensors frequently come into contact with biological fluids like saliva [2], urine [3], blood [4] or cell culture media [5]. Under these conditions the sensors must specifically detect their analytes like proteins or certain metabolites from a bulk of other substances in the surrounding medium. This requires sensor surfaces that are highly resistant to unspecific adsorption and at the same time provide a high number of binding sites for the specific analyte detection resulting in a good signal to noise ratio.

Additionally, for implantable biosensors or biosensors in blood contact the hemocompatibility must be carefully evaluated. Hemocompatibility can be constricted by undesired interactions between biomaterial and blood components leading to activation of coagulation, platelets and inflammatory response [6]. All these factors can cause harm to the patient and additionally lead to a negative influence onto the sensor signal [7,8].

Quartz crystal microbalance (QCM) sensors consist of piezoelectric quartz crystals. For electrical conductivity the sensor surface and electrodes are sputtered with thin layers of metals as gold, for example. To overcome the disadvantageous properties of metallic surfaces [9], frequently used sensor coatings include polysaccharides like sulfated dextran [10], tetraethylene glycol dimethylether (tetraglyme) [11] polydimethylsiloxane (PDMS) [12], and modified polyethyleneglycol and polypropyleneglycol derivates [13–16].

A few years ago the NCO-sP(EO-*stat*-PO) coating system was introduced as alternative to common coating systems for glass and silicon substrates [17]. NCO-sP(EO-*stat*-PO) is a six arm star shaped random copolymer of ethylene oxide and propylene oxide in a ratio 4:1 with terminal isocyanate groups. This system differs from conventional grafted linear PEGs in the hexafunctionality of the NCO-sP(EO-*stat*-PO) macromers that react and cross-link in water. This leads on the one hand to a high polymer segment density on the surface [18]. Moreover, the cross-linking enables the generation of layers that are thicker than monolayers which is extremely important for the long-time ability to resist cell adhesion [19]. Furthermore these terminal isocyanates are suitable functional groups for further modification with receptor molecules such as biotin [18], aptamers [20] or immobilized enzymes [21]. While covalent anchoring to the substrate provides a good stability for regeneration processes, the brush like structure of the star shaped polymers creates a kinetically energy barrier that hinders protein adsorption. Furthermore, the high surface coverage and the strong hydrophilic character of the polymer also contribute to resistance to cell and protein adhesion. In this study we have evaluated the applicability of NCO-sP(EO-*stat*-PO) to gold substrates and their hemocompatibility as auspicious coating material for gold sputtered blood sensors.

## 2. Experimental Section

### 2.1. QCM Sensor Platform

Quartz crystal microbalances are mass-sensitive sensor devices that can detect even very low mass deposition on sensor surfaces like proteins or biofilms. The attachment of masses to the sensor surface leads to changes in resonance frequency which can be electronically recorded. For our experiments we used a quartz crystal microbalance system called “FidgeType FgT1” [22–25]. The two-channel thermo controlled sensor platform was kept at constant of 37 °C for simulating in vivo conditions. The system uses an oscillator circuit and AT-cut quartz sensors (KVG Quartz Crystal Technology, Neckarbischoffsheim, Germany) with 8 mm diameter, 166 μm thickness and 10 MHz resonance frequency. The quartz sensors were sputtered with 2 nm chromium as adhesion promoting agent for the 100 nm thick gold layer.

### 2.2. Sensor Coating and Preparation

QCM sensors were first cleaned with acetone (Sigma Aldrich, Steinheim, Germany) for 1 min, then rinsed with deionized water and dried under a stream of nitrogen. The sensors were cleaned for 1 min in piranha solution (a 1:3 v/v solution of 30% hydrogen peroxide and concentrated sulphuric acid) and then rinsed with deionized water again. Next the sensors were again dried in a stream of nitrogen. To avoid pollution with airborne substances, the measurements with the uncoated gold sensors were performed within hours after the cleaning procedure.

Isocyanate (NCO) conjugated star shaped polymers (sP) of a statistical (*stat*) copolymer of ethylene oxide (EO) and propylene oxide (PO), NCO-sP(EO-*stat*-PO) are six arm star shaped molecules with terminal isocyanate groups. The backbone consists of a statistical copolymer of ethylene oxide and propylene oxide in a 4:1 ratio. The molecular mass of each arm is 2 kD. NCO-sP(EO-*stat*-PO) was synthesized according to procedures reported before [15,26]. Briefly, the star shaped hexafunctional alcohol was dried and reacted with freshly distilled isophorone-diisocyanate (IPDI) in a solvent free process at 50 °C for 5 days. Excess IPDI was removed by short path distillation, purity of the product was analyzed using size exclusion chromatography (SEC) and NMR.

For NCO-sP(EO-*stat*-PO) coating after the cleaning procedure, the sensor surfaces were treated with an aqueous solution of 10 mM cystamine (cystamine dihydrochloride, Sigma-Aldrich, St-Louis, USA) and allowed to react overnight at room temperature (in darkness and under argon atmosphere). Cystamine has two functional groups: amino-groups and thiol-groups. While the thiol-groups react with the gold on the sensor surface and provide covalent bonding, the amino-groups represent a suitable functional group for the following NCO-sP(EO-*stat*-PO) coating. After incubation overnight with cystamine the sensors were rinsed with double distilled water and treated for 15 min at RT with 0.5 M NaHCO_3_ (0.5 M aqueous solution of sodium hydrogen carbonate, pH 10). The treatment with this alkaline solution leads to the deprotonation of potential NH_3_^+^ groups to NH_2_ groups and facilitates the reaction with NCO-sP(EO-*stat*-PO). Tetrahydofuran (THF) was pre-distilled with activated carbon and potassium hydroxide, and finally dried with sodium benzophenone. NCO-sP(EO-*stat*-PO) was dissolved in this absolute water free THF in concentrations of 50 mg/mL. Polymerisation was started by the addition of 90 μL of double distilled water to aliquots of 10 μL THF dissolved NCO-sP(EO-*stat*-PO) resulting in final NCO-sP(EO-*stat*-PO) concentrations of 5 mg/mL. The samples were allowed to react for 5 min at RT before spincoating. NCO-sP(EO-*stat*-PO) films were generated by spincoating (Spi Supplies spincoater kw 4a, Spi Supplies, West Chester, PA, USA) for 45 s at 2,500 rpm (acceleration 500 rpm/s). For complete polymerisation, the NCO-sP(EO-*stat*-PO) coated sensors were stored overnight at RT in darkness.

### 2.3. Characterisation of the NCO-sP(EO-stat-PO) Coating

#### 2.3.1. Determination of Layer Thickness (Ellipsometry)

To determine the coating thickness of the spin coated NCO-sP(EO-*stat*-PO) films a customized imaging spectral ellipsometer from OMT (Optische Messtechnik Gmbh, Ulm, Germany) was used. Spectral ellipsometers utilize the transmission or reflectance of polarized light to determine film thickness (10 nm–100 μm). Measurements were performed at 3 different areas of the samples. The measurements with 3 different NCO-sP(EO-*stat*-PO) samples revealed coating thicknesses of 12.0 nm till 24.4 nm with standard deviations of 0.4 nm to 1.0 nm. At the edge of the measured samples the coating thickness was slightly higher than on the rest of the surface. This variation, the so called edge bead, is a result of the spin-coating process.

#### 2.3.2. Contact Angle Measurements

The wettability of the gold and NCO-sP(EO-*stat*-PO) surfaces (n = 2 for each group) was investigated with the DSA 10-Mk2 analysis system (Kruess, Hamburg, Germany). Ultrapure water droplets (Millipore, Schwalbach, Germany) with a volume of 1 μL are deposited on the crystal surface by means of an automated syringe application system and recorded by a high resolution camera, catching 25 frames per second. For each surface two samples were investigated by depositing three water droplets per sample. For the analysis of the drop profile the DSA software version 1.90.0.11 was applied. The drop shape recorded 5 s after surface contact is adapted to fit the mathematical model (tangent method type 1) which is then used to calculate the contact angle.

### 2.4. Blood Sample Preparation

Donation of human blood was approved by the local ethics committee of the University Hospital of Tuebingen and all donors gave their informed consent. Fresh human whole blood from healthy volunteers was collected in appropriate syringes for generation of platelet poor plasma (PPP) which was used for the QCM measurements. We utilized citrate monovettes containing 1.0 mL of 0.106 mol/L citrate solution (10.0 mL 9NC S-Monovette, Sarstedt, Nümbrecht-Rommelsdorf, Germany). The blood was centrifuged for 10 min at 1,500 ×g for separation of platelet poor plasma.

The hemocompatibility tests were performed with platelet rich plasma (PRP). Blood was collected from medication free, healthy volunteers (n = 3) by venipuncture with 1.4 mm Ø butterfly cannula from a large antecubital vein into sterile and pre-anticoagulated containers. The blood was anticoagulated with 1.0 IU/mL sodium heparin 25000 (Ratiopharm GmbH, Ulm, Germany), to avoid excessive coagulation activation. Then the collected blood was centrifuged at 150 ×g for 10 min at room temperature for separating platelet rich plasma (PRP). After that the PRP was carefully removed from the syringes. Cellcount was performed with a cellcounter (Micros 60 ABX Hematology, Montpellier, France) platelet count was found to be between 256.000 and 350.000 μL.

### 2.5. Protein Adsorption to the Sensor Surface

#### 2.5.1. QCM Measurements

For the QCM measurements of fibrinogen adsorption to the NCO-sP(EO-*stat*-PO) coatings, fibrinogen solution as well as 1 + 4 diluted human platelet poor plasma (PPP) was used. Fibrinogen from human plasma (No.F4883, Sigma-Aldrich) was dissolved in phosphate buffered saline (PBS, pH 7.4, Gibco, Invitrogen, Karlsruhe, Germany) in resulting concentrations of 0.5 mg/mL. PPP was likewise diluted with PBS in a 1 + 4 ratio.

Prior to the measurements, quartz crystals were installed in the “FidgeType FgT1” device and stable baselines were recorded with PBS-buffer at flow rates of 50 μL/min. After that the flow was changed from buffer to either fibrinogen solution or human PPP. The sensors were incubated with the respective reagents for 20 min (at a flow rate of 50 μL/min). During this time, proteins adsorb to the sensor surface. Subsequently loosely bound proteins were allowed to desorb from the sensors surface through rinsing with PBS for 30 min (flow 50 μL/min). After that the sensors were dismantled from the measuring device and prepared for ELISA tests for fibrinogen adsorption.

#### 2.5.2. ELISA Tests for Adsorbed Fibrinogen

After the QCM measurements, the dismantled sensors were tested with a modified ELISA technique for fibrinogen adsorption. Next to the QCM measurements the sensors were incubated for 30 min at room temperature with 4% PBS based paraformaldehyde (pH 7.4 Merck, Darmstadt, Germany). Afterwards, the sensors were washed with buffer (washing buffer pH 7.4, Candor biosciences, Weißensberg, Germany). For neutralisation of potential paraformaldehyde residues, the sensors were incubated for 30 min at room temperature in 500 mM glycine solution (pH 7.4, Sigma Aldrich). Then the sensors were washed with buffer again and incubated overnight at 4 °C in blocking solution (Candor blocking solution). After a further washing step, the sensors were incubated with adequate antibodies dissolved in low cross buffer (Candor low cross buffer, pH 7.4). Surface adsorbed fibrinogen was detected with a specific primary antibody (goat anti human fibrinogen F2506, Sigma Aldrich) and an adequate secondary antibody (donkey anti sheep IgG alkaline phosphatase A5187 Sigma Aldrich). Incubation with each antibody was performed for 2 h at room temperature. After every incubation step the sensors were rinsed five times with washing buffer, to remove unbound antibodies. Next the alkaline phosphatase conjugated secondary antibody was incubated for 5 min with Sigmafast™ *p*-nitrophenyl phosphate substrate (1 mg/mL *p*-nitrophenyl phosphate, Tris-buffered, pH 7.4, Sigma Alrich). By reaction with alkaline phospatase, this substrate develops a soluble yellow reaction product that can be measured at 405 nm. The absorption measurements were carried out with a multimode fluorescence and absorbance reader (Berthold Mithras LB 940, Berthold Technologies, Bad Wildbad, Germany)

### 2.6. Hemocompatibility Tests

#### 2.6.1. Sample Preparation and Incubation on the Rocking Platform

A total volume of approximately 30 mL of platelet rich plasma from one single donor was aliquoted into four samples each containing 6 mL of PRP. For baseline measurement (“t0”), the first blood sample (6 mL) from each donor was taken without contact to the according quartz specimens or the suspension culture plates. Three different blood donors participated in our experiments. PRP incubation was performed on a rocking platform (Polymax 1040, Heidolph, Schwabach, Germany) at 25 rpm for 60 min at 37 °C. After incubation, blood was pooled within the four wells of one group and collected in adequate syringes for the determination of activation markers. For each donor four NCO-sP(EO-*stat*-PO) coated sensors and four uncoated gold sensors were incubated with blood in a 24 well suspension culture plate (Cellstar^®^ No.662102, Greiner Bio-One, Kremsmünster, Austria). Background activation (ctrl.) was determined by the incubation of 4 empty wells with blood from each donor.

#### 2.6.2. Blood Sampling

After 60 min of incubation on the rocking platform, blood was collected in appropriate syringes containing 1.6 mg EDTA/ml blood (EDTA, ethylenediamine tetraacetic acid, 2.7 mL K3E S-Monovette, Sarstedt, Nümbrecht-Rommelsdorf, Germany) for cell counting, 1.0 mL of 0.106 mol/L citrate solution (10.0 mL 9NC S-Monovette, Sarstedt, Nümbrecht-Rommelsdorf, Germany) for thrombin-antithrombin-complex (TAT) measurement or 4.5 mL CTAD-Vacutainer (450 μL of 0,109 M, CTAD = citrate, theophylline, adenosine dipyridamole solution, REF 367599, Becton Dickinson GmbH, Heidelberg, Germany) for evaluation of β-thromboglobulin (β-TG) and platelet factor 4 (PF4) levels.

#### 2.6.3. Analysis of Activation Markers

The samples were immediately centrifuged and Plasma of the blood samples was then aliquoted in 200 μL samples and shock frozen in liquid nitrogen with subsequent storage at −80 °C for further investigations. Changes in markers of coagulation and complement activation as well as blood cell release factors were measured by commercially available ELISA kits. Samples were analysed for ß-thromboglobulin (Asserachrom ß-TG, Diagnostica Stago, Asnieres, France) and platelet factor 4 (PF4, Asserachrom) as platelet activation markers, and thrombin-antithrombin-III complex (Enzygnost TAT micro, Dade Behring, Schwalbach Germany) to evaluate activation of plasmatic coagulation.

#### 2.6.4. Scanning Electron Microscopy

After 60 min of incubation with PRP on the rocking platform, the sensors were gently rinsed with PBS for removal of non adherent platelets. Afterwards the quartz crystals were incubated overnight at 4 °C in 2% PBS based glutaraldehyde solution. Next, the quartz crystals were washed again with PBS to remove residual glutaraldehyde. After that, the remaining water was removed from the samples using 40% to 100% of ethanol (Merck, Darmstadt, Germany) in ascending concentrations. Finally, all samples were critical point dried (CPD), sputtered with gold palladium, and then analysed using SEM (scanning electron microscopy, Cambridge Instruments, Cambridge UK, type 250 MK2).

#### 2.6.5. Statistical Procedure

Frequency shifts and absorbance were expressed in the graphs as arithmetic mean (M) values with ± standard deviation (SD). Statistical analysis was performed with the software BIAS for Windows™ Version 9.06 (Epsilon Verlag, Frankfurt, Germany). Data were tested for normal distribution by Kolmogorow-Smirnow test. Homogeneity of variances was tested by Bartlett’s test and multiple comparison with Scheffé’s method. Differences between groups were calculated by univariate analysis of variance. Values of p < 0.05 were considered to be significant.

## 3. Results and Discussion

### 3.1. QCM Measurements and ELISA Tests for Adsorbed Fibrinogen

**Figure 1.**
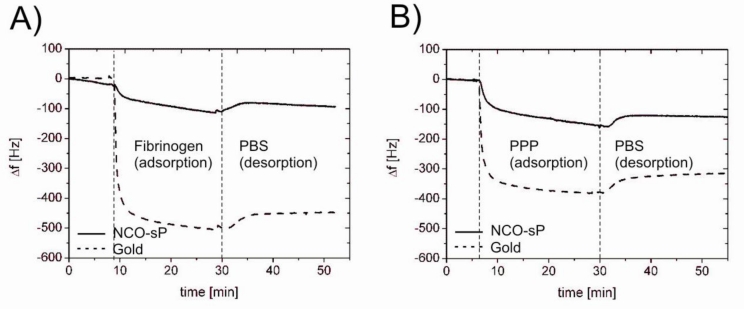
Protein adsorption measured by QCM with fibrinogen solution (0.5 mg/mL, in PBS, pH 7.4) (**A**) and diluted human plasma (1 + 4 diluted in PBS, pH 7.4) (**B**).

**Figure 2.**
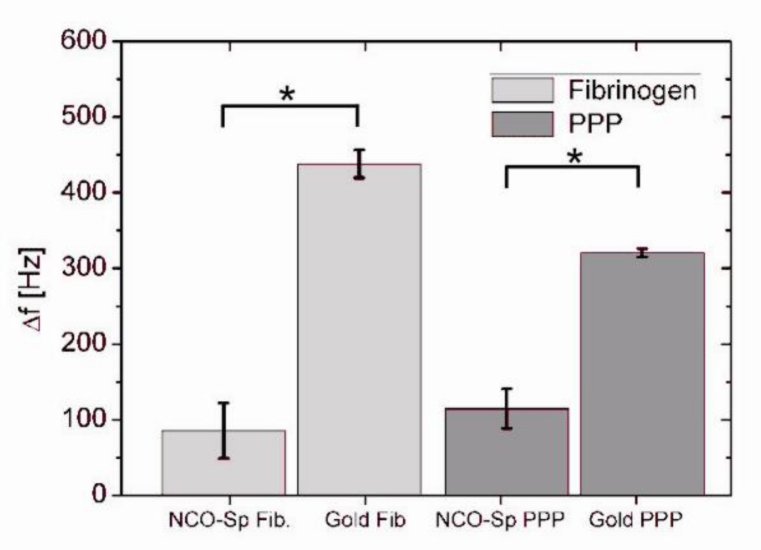
QCM frequency changes during protein adsorption measurements. NCO-sP(EO-*stat*-PO) coated and uncoated gold sensors were incubated in the QCM device with fibrinogen solution and 1 + 4 diluted human PPP (n = 3, for each Group). Differences between groups were calculated by univariate analysis of variance. Values of p < 0.05 were considered as significant and marked with *.

NCO-sP(EO-*stat*-PO) coated and uncoated QCM sensors responded to contact with PBS based fibrinogen solution and 1 + 4 diluted human plasma with a drop in resonance frequency. Exemplary measuring curves of the respective experiments are shown in Figure 1. In the first phase of the reaction after the reagent has reached the measuring chamber protein adsorption to the sensor surface took place. The adsorption was accompanied by a decrease of the resonance frequency. Rinsing with buffer in the following step led to the removal of loosely bound proteins and a following increase of resonance frequency. For measurements with 0.5 mg/mL fibrinogen solution the average drop of resonance frequency was 85 Hz ± 37 Hz for NCO-sP(EO-*stat*-PO) coated sensors and 438 Hz ± 18 Hz for the uncoated gold sensors (Figure 2). During the measurements with 1 + 4 diluted human PPP, NCO-sP(EO-*stat*-PO) coated sensors revealed frequency shifts of 115 Hz ± 26 Hz. Frequency changes for the gold sensors in this group were 320 Hz ± 6 Hz (Figure 2).

**Figure 3.**
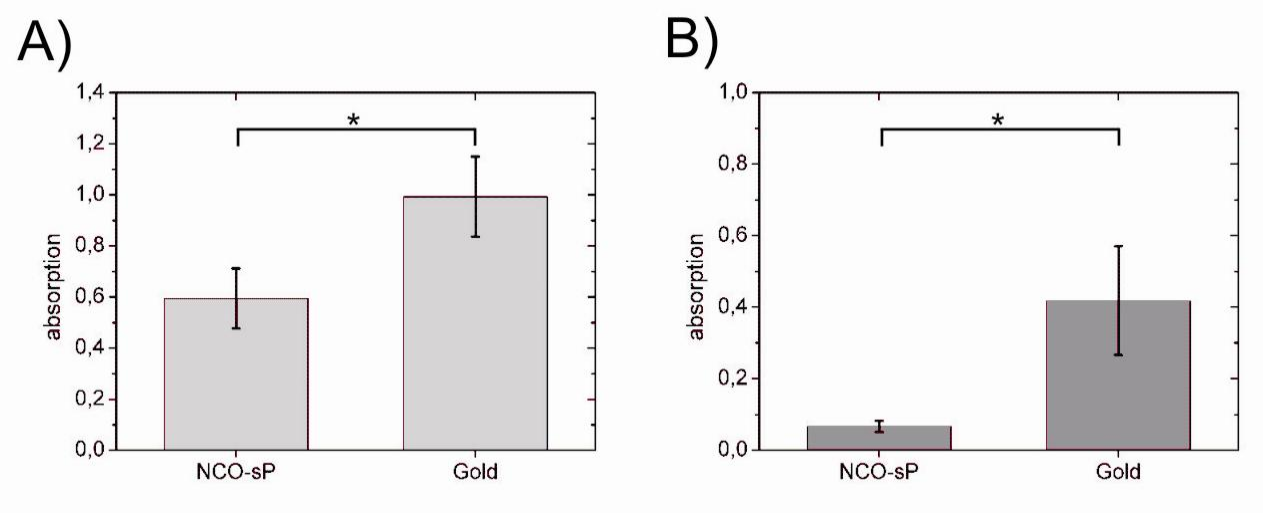
Modified ELISA tests for fibrinogen adsorption from 0.5 mg/mL fibrinogen solution (**A**) und 1 + 4 diluted human PPP (**B**). Differences between groups were calculated by univariate analysis of variance (n = 3 for each group). Values of p < 0.05 were considered as significant and marked with *.

In 0.5 mg/mL fibrinogen solution as well as in 1 + 4 diluted human plasma, there was a significant difference between NCO-sP(EO-*stat*-PO) and uncoated sensors (p < 0.05). In the ELISA tests for adsorbed fibrinogen (Figure 3), fibrinogen adsorption was compared within each of the groups, 0.5 mg/mL fibrinogen solution and 1 + 4 diluted PPP. In comparison with the uncoated gold sensors, fibrinogen adsorption was significantly reduced on NCO-sP(EO-*stat*-PO) coated sensors in both groups (p < 0.05).

Most biomedical blood contacting devices are made of materials that were not specifically designed for a medical application, such as various metals like stainless steel [27] and nitinol (used for stents) [28] or gold used as QCM sensor coating. Since it is technically not possible to replace all of these materials, surface coatings with improved hemocompatibility are of growing importance [29]. Resistance to platelet adhesion and to unspecific protein adsorption as well as preferably low activation of blood coagulation, are the most important requirements for a hemocompatible coating. Finally, the same coatings that are advantageous for blood contacting devices may also be suitable for biosensors in blood contact. In this application the coatings may prevent unspecific adsorption of cells and proteins [30] as well, which could otherwise lead to a loss of sensor signal over time [11]. The overall protein concentration in human plasma is about 60 to 80 mg/mL [31]. The resulting protein concentrations in 1 + 4 diluted PPP are 12 mg/mL. The average fibrinogen content of human plasma is 0.15 to 0.35 mg/mL [32], so that in the PBS based fibrinogen solution there is still twice the amount of fibrinogen normally found in undiluted PPP. Fibrinogen is the most important protein for undesired platelet adhesion in cardiopulmonary bypass procedures [33].

Previous publications concerning the protein and cell repellent properties of PEGylated terpolymers have found that fibrinogen and platelet repellent properties improve with the increasing amount of PEGylated compounds like poly(ethylene glycol) methyl ether methacrylate (PEGMA) being higher than 15% of the overall mass of the polymer [34]. The coating of hydrophobic polymers like polystyrene with PEGs leads to improved protein resistance even at an incomplete surface coverage [35]. Best results for protein resistance can be achieved with coatings of preferably high grafting density [36], which must be carefully concerted to molecular weights of the used PEGs. There are different notions about the minimum chain length for the generation of protein repellent surfaces [37,38], and for the optimal surface density of the individual chains.

Compared to linear PEGs, at a similar chain length and molecular weight, the star shaped polymer brushes variants have a much higher polymer density, resulting in greater steric repulsive forces against adsorbing proteins [39]. In contrast to polymer brushes alkanethiolate SAMs are more prone to layer defects [40] and have a decreased stability against oxidation processes [41]. While oxidation processes hamper the long term functionality of the protein resistant coating, layer defects are known to provide vulnerable spots for undesired protein adsorption [42]. Since SAMs consist of individual alkanethiolate chains with functional groups like PEO, but usually do not have crosslinkers between the individual chains, the layer defects revealing uncoated spots on the substrate surface cannot be closed by cross reaction with adjacent chains.

Unsworth *et al*. [43] investigated the effects of PEO chain density on protein resistance in SAMs. In their fibrinogen adsorption experiments they found that with optimal chain size of 750 g/mol at the ideal surface density of 0.5 chains/nm^2^ protein adsorption was reduced as much as 80% compared to uncoated gold surfaces. In our experiments with 0.5 mg/mL fibrinogen solution, judging from QCM data, we also found a 80% reduction of fibrinogen adsorption on NCO-sP(EO-*stat*-PO) coated surfaces. In the experiments with 1 + 4 diluted PPP we detected reductions of overall protein adsorption of 64%. Since in our experiments the resistance against fibrinogen adsorption is higher than the resistence against overall plasma protein adsorption we have to assume that there are other proteins in plasma that adsorb more easily to the NCO-sP(EO-*stat*-PO) coating than fibrinogen.

At this point the size of the proteins also comes into play. Sofia *et al*. [44] have shown that their star shaped PEGs had a good resistance against albumin and fibrinogen adsorption but were prone to adsorbing cytochrome c. The explanation for that was seen in the fact, that cytochrome c is smaller in size than albumin and fibrinogen and can therefore adsorb in the gap between the polymer brushes. This problem could be overcome in increasing the concentration and grafting density of the polymer brushes

Scott *et al*. [45] used a dip coating strategy to produce a polyethylene glycol micro gel coating for glass coverslips and polyethylene terephthalate discs. For the formation of microgels they used a PEG-octavinylsulfone (PEG-OVS) substrate with bovine serum albumin (BSA) or PEGoctaamine (PEG-OA) as crosslinkers. Long term resistance against cell adhesion was tested under cell culture conditions over several days. Protein adhesion to the sensor surfaces was assessed by QCM measurements. The PEG microgels revealed increased resistance to both cell and protein adhesion. Our QCM measurements are in good accordance to the findings of Scott *et al*. however in our measurements not all of the initially adsorbed fibrinogen can be removed by the consecutive washing with buffer.

To prove our coating technique for NCO-sP(EO-*stat*-PO) and to exclude unspecific adsorption from other plasma proteins than fibrinogen, in further studies with greater sample size the resistance to adsorption of proteins like albumin and immunoglobulins should also be studied.

### 3.2. Hemocompatibility Tests

**Figure 4.**
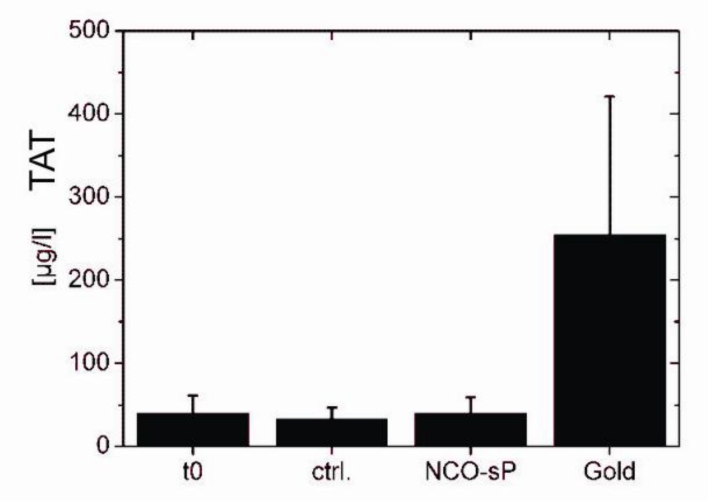
Plasma concentration of coagulation marker thrombin antithrombin complex (TAT). Before (t0) and after 60 min of PRP incubation on the rocking platform. Samples without sensors (ctrl.), with NCO-sP(EO-*stat*-PO) coated sensors (NCO-sP(EO-*stat*-PO)) and uncoated gold sensors (Gold). For each group 3 samples (n = 3) were tested.

**Figure 5.**
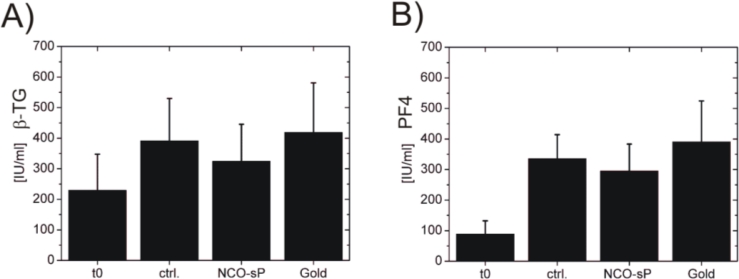
Plasma concentrations platelet activation markers. ELISA tests for β-thromboglobulin (**A**) and platelet factor 4 (**B**). Before (t0) and after 60 min of PRP incubation on the rocking platform, Sample without sensors (ctrl.), with NCO-sP(EO-*stat*-PO) coated sensors (NCO-sP(EO-*stat*-PO)) and uncoated gold sensors (Gold) each group 3 samples (n = 3) were tested.

In the hemocompatibility tests for plasmatic coagulation activation represented by TAT generation (Figure 4) the baseline (t0) measurements were 40.26 μg/L ± 21.5 μg/L, the control measurements (ctrl.) had values of 32.87 μg/L ± 14.06 μg/L. Between NCO-sP(EO-*stat*-PO) with 39.49 μg/L ± 19.66 μg/L and gold with 254.68 μg/L ± 166.19 μg/L, there were numerically though not statistically significant differences. Testing for markers of platelet activation (Figure 5). PF4 revealed values of 89.20 IU/mL ± 42.79 IU/mL for baseline measurements (t0) and 335.53 IU/mL ± 78.58 IU/mL for controls (ctrl.) NCO-sP(EO-*stat*-PO) coated sensors resulted in values of 295.39 IU/mL ± 88.03 IU/mL, whereas the gold sensors achieved values of 390.99 IU/mL ± 133.41 IU/mL.

Supplementary to PF4, β-TG was used as a further marker for platelet activation. The β-TG values were 229.39 IU/mL ± 118.11 IU/mL for baseline measurements (t0) and 391.63 IU/mL ± 138.41 IU/mL for controls of background activation (ctrl.). Sensors with NCO-sP(EO-*stat*-PO) coating had values of 324.56 IU/mL ± 121.08 IU/mL and the uncoated gold sensors revealed values of 418.65 IU/mL ± 162.43 IU/mL.

With the hemocompatibility tests coagulation activation, quantified by TAT generation was numerically, though not statistically significantly reduced in NCO-sP(EO-*stat*-PO) coated sensors. Comparatively low plasmatic coagulation activation may also be attributed to the protein repellent properties of NCO-sP(EO-*stat*-PO) [46].

Similar outcomes for plasmatic coagulation activation through metallic surfaces have also been found by Anderson *et al*. [47] who compared surface associated coagulation activation with human PPP in QCM sensors coated with different polymers like polystyrene and polyurethane, Heparin as well as uncoated titanium sensors. Measurements were quantified through frequency and dissipation shifts on the QCM device. They found the highest coagulation activation in uncoated titanium, with average coagulation activation for the different polymers and heparin coated sensors being the least thrombogenic.

Hulander *et al*. [9] also investigated the procoagulant properties of different noble metals such as gold, silver, palladium and titanium and compared them with silver containing Bactiguard coating. In their hemocompatibility tests they found that besides pure surface chemistry also the nanotopograhy of the surface coating seems to have a major influence on coagulation activation and the amount of adsorbed fibrinogen.

Within our studies, in platelet activation, represented by β-TG and PF 4, there were no significant differences between controls (ctrl.), NCO-sP(EO-*stat*-PO), and gold. For improved validity of the hemocompatibility tests further studies should involve a bigger sample size and a more sophisticated *in vitro* test system which can hold larger blood volumes like the *in vitro* closed loop system first introduced by Chandler [48], which went through further development and is also routinely used in our lab [49]. As a further advantage NCO-sP(EO-*stat*-PO) coated Chandler Loops with the respective uncoated controls would also represent a larger surface for blood activation processes. The NCO-sP(EO-*stat*-PO) coated sensors represent a relatively small surface compared to the background of the multi well plates that were used for the experiments. Admittedly for the extremely sensitive QCM sensors even marginal improvements towards the optimal surface coating are of importance.

Compared to the studies of Scott *et al*. we did not perform experiments for long term resistance to cell adhesion under cell culture conditions, but the hemocompatibility tests can also confirm that our NCO-sP(EO-*stat*-PO) polymers revealed only marginal platelet adhesion to the sensor surface. Resistance to unspecific platelet adhesion is one of the major requirements of a whole blood contacting biosensor, especially when the sensor is applied in real time measurements of blood parameters like the detection of individual coagulation factors [50]. For long term measurements with implantable biosensors resistance against endothelial cell and fibroblast adhesion should also be tested. In this aspect PEG polymer brushes have an advantage over other PEG based self assembling monolayers that exhibit also short term cell and protein resistance but get seeded with cells in the course of days [51].

### 3.3. Contact Angle Measurements

NCO-sP(EO-*stat*-PO) coating of the sensors led to increased surface wettability. After coating the contact angles changed from 69° ± 1.4° for the uncoated gold sputtered quartz sensors to 22° ± 7° for the NCO-sP(EO-*stat*-PO) coated sensors (Table 1). The high hydrophilicity of NCO-sP(EO-*stat*-PO) and other PEGs is seen as one of the contributing factors to protein resistance and hemocompatibility [52,53]. The results of the determination of layer thickness are shown in Table 2.

### 3.4. Scanning Electron Microscopic Images

**Figure 6.**
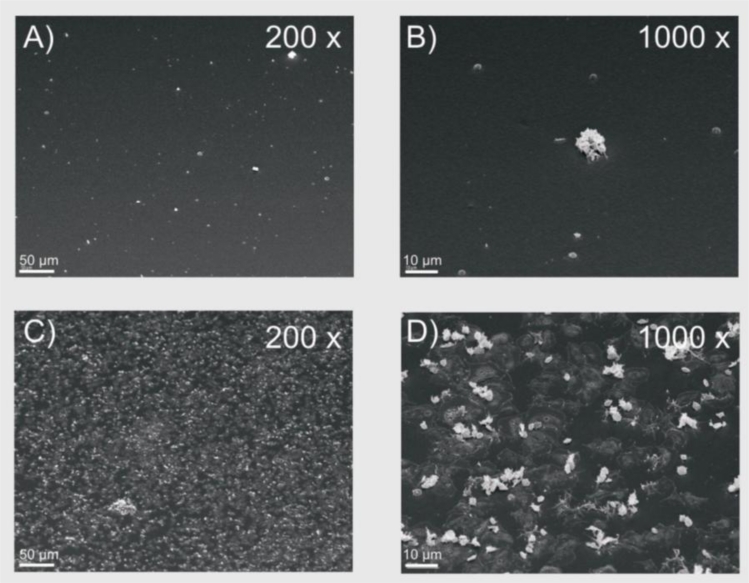
Scanning electron microscopic images of NCO-sP(EO-*stat*-PO) coated sensors (**A**) and (**B**) and uncoated gold sensors (**C**) and (**D**).

Scanning electron microscopic images served as visualization of the sensor surfaces after exposure to PRP. The SEM images (Figure 6) show only a few platelets attached to the NCO-sP(EO-*stat*-PO) coated sensors; opposed to the uncoated gold sensors, which were densely covered with adherent platelets. Although these data were not quantified, a lesser extend of platelet deposition on the NCO-sP(EO-*stat*-PO) sensors is in consistence with the lower fibrinogen adsorption on the NCO-sP(EO-*stat*-PO) sensors, and indicates an increased resistance against unspecific cell adhesion. Since protein adsorption is respected to be an essential precondition for the following cell adhesion QCM, ELISA and SEM data are in good accordance with each other. This seems to be convenient with earlier studies that have revealed, that pre-adsorbed fibrinogen facilitates the following platelet adhesion [54,55].

## 4. Conclusions

Within this study we demonstrated hemocompatibility, cell- and protein-repellent properties of NCO-sP(EO-*stat*-PO) for gold QCM sensor coatings. In applications in which the use of metallic compounds is indispensable like stents or metallic biosensors, the adaption of NCO-sP(EO-*stat*-PO) coating may help to reduce unspecific protein adsorption, cell attachment and possibly thrombo-embolic complications. In future modifiable NCO-sP(EO-*stat*-PO) coatings of biosensors may become an alternative to the direct attachment of the recognition elements to the sensor surface and therefore may prolong sensor lifetime and sensitivity.

## Figures and Tables

**Table 1.** Static contact angle measurements on uncoated gold sensors and NCO-sP (EO-*stat*-PO) coated sensors.

**Surface**	**Water contact angle[°] mean ± standard deviation**
Gold	69 ± 1.4
NCO-sP(EO-*stat*-PO) (5 mg)	22 ± 4.7

**Table 2.** Determination of layer thickness and homogeneity of the coatings with ellipsometry.

**Sample**	**Layer thickness [nm] mean ± standard deviation**
NCO-sP(EO-*stat*-PO) sample1	19.6 ± 0.4
NCO-sP(EO-*stat*-PO) sample2	24.4 ± 1.0
NCO-sP(EO-*stat*-PO) sample3	12.0 ± 0.8
